# Inhibition of the foot-and-mouth disease virus subgenomic replicon by RNA aptamers

**DOI:** 10.1099/vir.0.067751-0

**Published:** 2014-12

**Authors:** Sophie Forrest, Zoe Lear, Morgan R. Herod, Martin Ryan, David J. Rowlands, Nicola J. Stonehouse

**Affiliations:** 1 School of Molecular and Cellular Biology, Faculty of Biological Sciences and Astbury Centre for Structural Molecular Biology, University of Leeds, Leeds LS2 9JT, UK; 2 Biomedical Sciences Research Complex (BSRC), School of Biology, University of St Andrews, North Haugh, St Andrews, Fife KY16 9ST, UK

## Abstract

We have previously documented the inhibitory activity of RNA aptamers to the RNA-dependent RNA polymerase of foot-and-mouth disease virus (3D^pol^). Here we report their modification and use with a subgenomic replicon incorporating GFP (pGFP-PAC replicon), allowing replication to be monitored and quantified in real-time. GFP expression in transfected BHK-21 cells reached a maximum at approximately 8 h post-transfection, at which time change in morphology of the cells was consistent with a virus-induced cytopathic effect. However, transfection of replicon-bearing cells with a 3D^pol^ aptamer RNA resulted in inhibition of GFP expression and maintenance of normal cell morphology, whereas a control aptamer RNA had little effect. The inhibition was correlated with a reduction in 3D^pol^ (detected by immunoblotting) and shown to be dose dependent. The 3D^pol^ aptamers appeared to be more effective than 2′-*C*-methylcytidine (2′CMC). Aptamers to components of the replication complex are therefore useful molecular tools for studying viral replication and also have potential as diagnostic molecules in the future.

## Introduction

Foot-and-mouth disease is an acute, systemic vesicular disease in cloven-hoofed animals resulting from infection by foot-and-mouth disease virus, FMDV (reviewed by Jamal & Belsham, 2013). Major outbreaks in Taiwan (in 1997) and the UK (in 2001) had serious consequences for agriculture and tourism (Thompson *et al.*, 2002; Yang *et al.*, 1999), with losses to the UK estimated in excess of £8 billion. The disease remains endemic in many parts of Asia, South America, Africa and the Middle East, including countries bordering on Europe, such as Turkey. Although severe trade restrictions are imposed on countries where the disease is endemic, outbreaks do occur elsewhere. The problem with controlling the spread of the disease is mainly due to high infectivity and transmissibility of the virus, and is complicated by the ability of the virus to develop an asymptomatic carrier state.

Like other picornaviruses, the FMDV genome is a positive-stranded RNA molecule comprising a large single ORF flanked by a long 5′ untranslated region (UTR), a short 3′UTR and a poly(A) tail. The 5′UTR contains at least five domains, including an internal ribosomal entry site (IRES), which enables cap-independent translation of the viral proteins (Belsham & Brangwyn, 1990), a poly(C) tract of unknown function and a *cre* (*cis*-acting replication element), involved in the initiation of RNA replication. The ORF comprises four major regions (L^pro^, P1, P2 and P3) and is translated into a single polyprotein, which then undergoes processing. L^pro^ is the protease responsible for cleavage of eIF4G and so inhibiting host protein synthesis. The P1 region encodes the four structural proteins required for the formation of the capsid. The P2 and P3 regions encode non-structural proteins responsible for modification of the host cell and assembling the replication machinery. Among these, the RNA-dependent-RNA polymerase (RdRp) 3D^pol^, its precursor 3CD and the primer fragment 3B (also known as VPg) are known to be key proteins in viral RNA replication, although further precursor proteins are also likely to be involved. There are several crystal structures of 3D^pol^, including a structure with the 3B protein primer fragment (Ferrer-Orta *et al.*, 2004, 2006, 2009). The presence of three encoded 3Bs (Nayak *et al.*, 2005) and the error-prone nature of FMDV 3D^pol^ (Domingo *et al.*, 2002) are thought to contribute to the ability of the virus to establish states of fast replication but also carrier states with reduced replication.

Much of our knowledge about picornaviral replication comes from studies with poliovirus (PV). However, there are several interesting differences between PV and FMDV, including the presence of three tandem copies of 3B (VPg) in the FMDV genome and the size and complexity of the 5′UTR. FMDV has a very fast replication rate (Domingo & Holland, 1997; Alexandersen et al., 2002) and it is likely that some of these unusual properties of the genome are responsible for this phenotype. Because of the economic importance and highly infectious nature of FMDV, work with the virus is restricted to high containment facilities; however, subgenomic replicons can be safely used to study many aspects of virus replication. The pGFP-PAC replicon employed here was modified from the pT7Rep construct (McInerney *et al.*, 2000), a cDNA of FMDV O_1_K strain in which L^pro^ and most of the structural proteins had been replaced with a chloramphenicol acetyltransferase gene. This new construct encodes the full L^pro^ protein and fragments of the 1A and 1D proteins flanking a GFP puromycin *N*-acetlytransferase (GFP/PAC) reporter gene cassette, which allows replication to be monitored in real time (Tulloch *et al.*, 2014).

We have previously selected and characterized RNA aptamers that can inhibit 3D^pol^ activity *in vitro* (Ellingham *et al.*, 2006; Bentham *et al.*, 2012). RNA aptamers are isolated by systematic evolution of ligands by exponential enrichment (SELEX) and bind their target molecules with high affinity and specificity. Modification of the aptamers by inclusion of 2′F pyrimidines, for example, increases stability (Yazbeck *et al.*, 2002; Osborne & Ellington, 1997). Without modifications to the sugar–phosphate backbone, the half-life of RNA molecules is minutes, making both experimental application and evaluation of therapeutic potential impossible. Several modifications have therefore been incorporated into aptamers undergoing clinical trials and in the development of Macugen, an anti-VEGF (anti-vascular endothelial growth factor) aptamer, the first commercially available aptamer therapeutic (Green *et al.*, 1995; Ruckman *et al.*, 1998; Ng et al., 2006). Furthermore, fluorophores can be added to the 3′ or 5′ end of the molecule to facilitate detection in cells.

We report here the use of chemically modified (2′F Cy3) 3D^pol^ RNA aptamers as tools to investigate replication of the pGFP-PAC replicon. We showed that the modified aptamers remained able to inhibit 3D^pol^ *in vitro* and demonstrated (by live cell imaging and immunoblotting) their ability to effectively inhibit replication.

## Results

### Modified aptamers inhibit 3D^pol^ *in vitro*

**Fig. 1.**
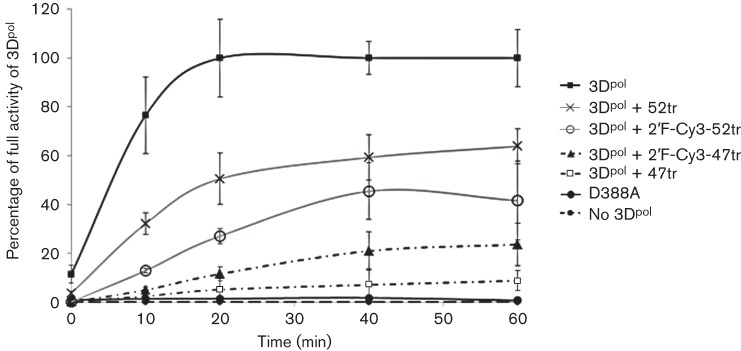
Effect of aptamers on the *in vitro* elongation activity of 3D^pol^ by incorporation of [^32^P] UTP. The 3D^pol^ active site mutant D388A was included as a negative control. The effects of pre-incubation with 2′-fluoro- and Cy3-modified aptamers (2′F-Cy3-47tr and 2′F-Cy3-52tr) and unmodified aptamers 47tr and 52tr (4 µM, 20 min) are also shown. Data are mean±se of three experiments. Statistical analyses comparing the effects of each aptamer to 3D alone were conducted using a *t-*test (*P*<0.01).

Previously, we characterized RNA aptamers selected to FMDV 3D^pol^ that were able to inhibit the activity of the enzyme *in vitro* (Ellingham *et al.*, 2006). The most promising of the aptamers were termed 47 and 52. These shared 75 % similarity in the random, selected region and were also active when truncated to eliminate the primer binding regions (termed 47tr and 52tr). We went on to show that 3D^pol^ could form RNA–protein fibrils *in vitro* (the products of an elongation assay) and that 47tr was able to inhibit their formation (Bentham *et al.*, 2012). These aptamers were selected without chemical modification and were therefore very susceptible to degradation both in serum and in cells. The incorporation of modified nucleotides into RNAs has been shown to increase stability; however, such modifications can alter RNA conformation. One of the least bulky substituents is fluorine at the 2′ position of ribose. In order to evaluate if the 3D^pol^ aptamers would tolerate such modifications and retain inhibitory activity we resynthesized the aptamers, incorporating 2′F and 5′Cy3, and performed elongation assays, monitoring [^32^P]UTP incorporation with a poly(A) template and oligo(dT) primer. The 3D^pol^ was able to incorporate radiolabelled UTP over time under these conditions and both the unmodified (47tr and 52tr) and modified (2′F-Cy3-47tr and 2′F-Cy3-52tr) aptamers were inhibitory (*P***<**0.01), as shown in Fig. 1. The 3D^pol^ active site mutant (D388A) was included as a negative control.

### Localization of aptamers in BHK-21 cells

**Fig. 2.**
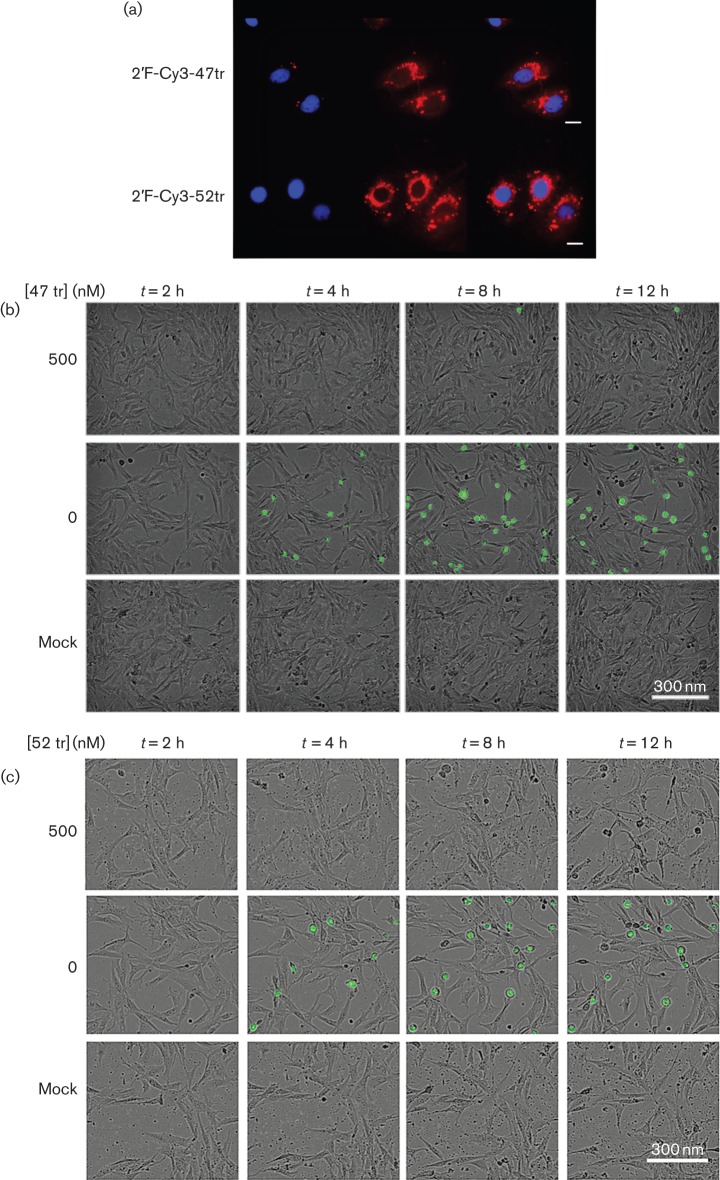
Aptamers can be detected in BHK-21 cells. (a) Cells grown on glass coverslips were transfected with 50 nM 2′F-Cy3-47tr or 2′F-Cy3-52tr aptamer (red). At 24 h post-transfection, cells were fixed, stained (DAPI) and imaged using an upright confocal microscope (Zeiss). Bars, 10 µm. (b, c) Cells were seeded and maintained for up to 24 h in 10 % FCS/DMEM at 37 °C, 5 % CO_2_ prior to transfection with 1 µg pGFP-PAC replicon RNA in the presence or absence of 2′F-Cy3-47tr (b) or 2′F-Cy3-52tr (c) at final concentrations of 500 nM. An IncuCyte was used to take nine images of every well, every 30 min. A selection of images at *t* = 2, 4, 8 and 12 h post-transfection are shown, as a merge of fluorescent and phase-contrast images, with mock-treated cells as a control.

To confirm that the modified aptamers could be delivered into mammalian cells, samples of both 2′F-Cy3-47tr and 2′F-Cy3-52tr were transfected into BHK-21 cells. Cells were stained with DAPI, and the presence of the Cy3 fluorophore allowed direct visualization of the RNA. Aptamers were detected throughout the cytoplasm but concentrated in the perinuclear region, for reasons currently unknown, as shown in Fig. 2(a).

### GFP expression as a measure of replication in the presence and absence of RNA aptamers

The inclusion of a GFP-PAC reporter protein within the pGFP-PAC replicon allows for the detection of replication over time using an IncuCyte FLR (Tulloch *et al.*, 2014). In cells transfected with replicon RNA, GFP expression could be detected within 4 h, with cytopathic effects becoming evident, as shown in Fig. 2(b, c) and the supplementary movies (available in the online Supplementary Material). However, in cells transfected with 2′F-Cy3-47tr and 2′F-Cy3-52tr aptamers, inhibition of expression was correlated with the loss of cytopathic effects, with the cells continuing to grow normally for the remainder of the experiment (Fig. 2b, c).

**Fig. 3.**
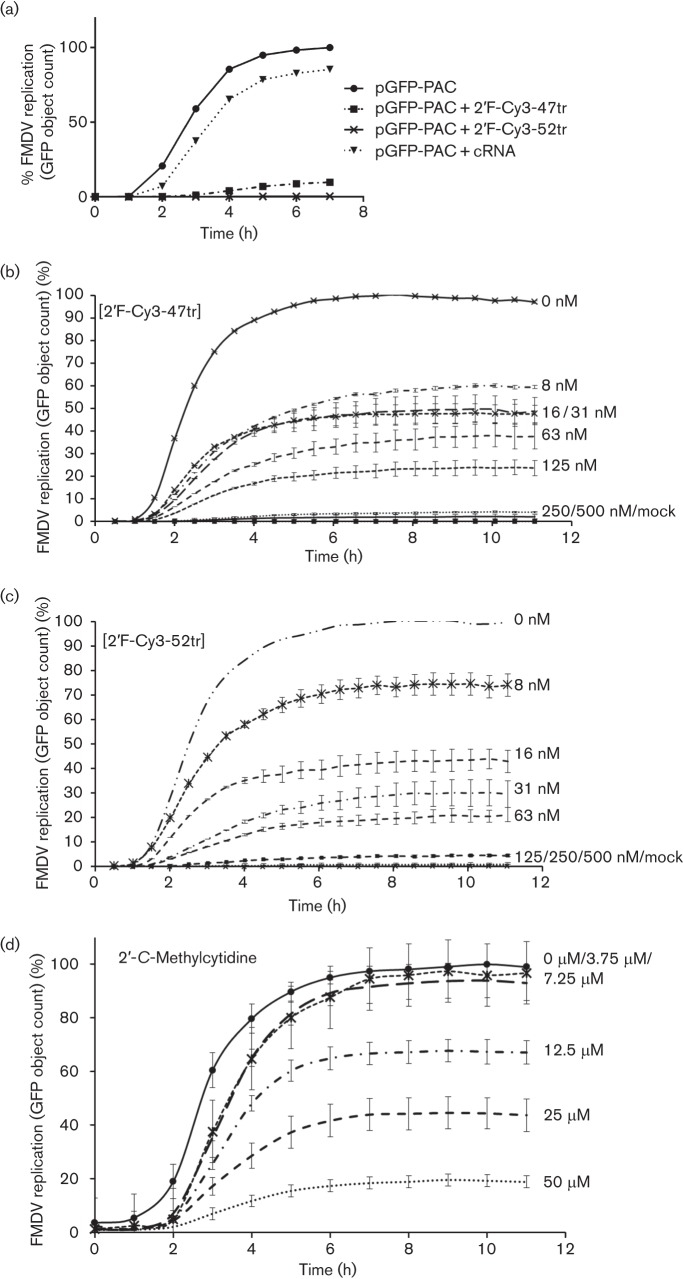
Quantification of pGFP-PAC replicon GFP expression over time in the presence and absence of aptamer. BHK-21 cells were seeded and maintained for 24 h in 10 % FCS/DMEM at 37 °C, 5 % CO_2_ prior to transfection with 1 µg pGFP-PAC replicon RNA. GFP expression was monitored using an IncuCyte FLR microscope. Data are presented as mean green object count of the pGFP-PAC replicon over up to 12 h from the point at which GFP expression is first detected. (a) GFP expression with 2′F-Cy3-47tr, 2′F-Cy3-52tr or cRNA at final concentrations of 500 nM. GFP expression with increasing concentrations (0 to 500 nM) of 2′F-Cy3-47tr (b) or 2′F-Cy3-52tr (c). (d) 2′-CMC (0–50 µM). Shown is the mean object count from a collection of nine images, allowing se to be calculated.

In addition to direct visualization, it was also possible to quantify levels of GFP expression over time using the approach above. These data confirmed that expression of GFP reached a maximum after 6–8 h (Fig. 3a and supplementary movies). Inclusion of 2′F-Cy3-47tr or 2′F-Cy3-52tr (500 nM) resulted in a dramatic reduction in GFP expression, whereas the control aptamer (cRNA, selected to the unrelated protein, IL-17A) had little effect (Fig. 3a). The relatively small reduction in GFP expression could be the result of co-transfection. It was further demonstrated that the effects of the 2′F-Cy3-47tr and 2′F-Cy3-52tr aptamers were dose dependent (Fig. 3b, c), with almost total inhibition of GFP expression at 125 nM for 2′F-Cy3-52tr and 250 nM for 2′F-Cy3-47tr. As a control, cells were also treated with 2′CMC, resulting in dose-dependent inhibition of GFP expression, by up to 80 % at 50 µM (Fig. 3d) (Stuyver *et al.*, 2006). Although it appeared that this compound was only effective at a much higher concentration, it should be noted that the method of delivery of the 2′CMC was different to that of the aptamers.

**Fig. 4.**
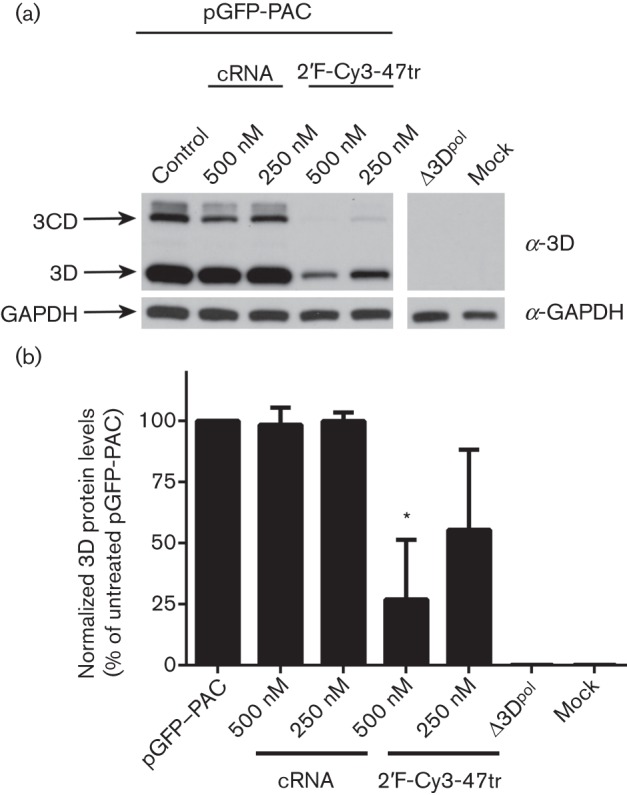
Effect of aptamers on 3D^pol^ expression in BHK-21 cells transfected with pGFP-PAC replicon. Cell extracts were prepared at 4 h post-transfection. (a) Aliquots were analysed by SDS-PAGE and immunoblotting for the presence of FMDV 3D^pol^ and cellular glyceraldehyde 3-phosphate dehydrogenase (GAPDH). Cells transfected with a Δ3D^pol^ replicon (pGFP-PAC-Δ3D), mock-transfected or transfected with cRNA were included as controls. (b) Densitometry was conducted using ImageJ imaging analysis software on triplicate experiments for 3D^pol^ expression, normalized to GAPDH. Data show mean values with sd (*n* = 3) and statistical analysis performed using two-tailed paired *t*-test (* = *P*<0.05).

To confirm that the reduction in GFP expression correlated with inhibition of replicon replication, 3D^pol^ expression over time was investigated by immunoblotting. Fig. 4 shows that 3D^pol^ could be detected at 4 h post-transfection and that a band corresponding to the expected size of the precursor protein 3CD was also evident. The 3D^pol^ and 3CD bands were absent in the lane corresponding to the control Δ3D^pol^ (a replicon including a deletion of most of the 3D gene), as expected. In the presence of 2′F-Cy3-47tr, the 3D^pol^ level was greatly reduced (to 26.9±24.4 and 55.49±32.8 % with 500 and 250 nM, respectively, *P*<0.05). In addition, there seemed to be a reduction in the 3CD translation product with 2′F-Cy3-47tr. Very little reduction was seen with the cRNA (to 98.5±6.9 and 99.9±3.5 % for 500 and 250 nM, respectively). The inhibition attributable to the specific aptamer was therefore much more pronounced, and appeared to be dose dependent.

### Modified aptamers appear to be specific inhibitors of replication

**Fig. 5.**
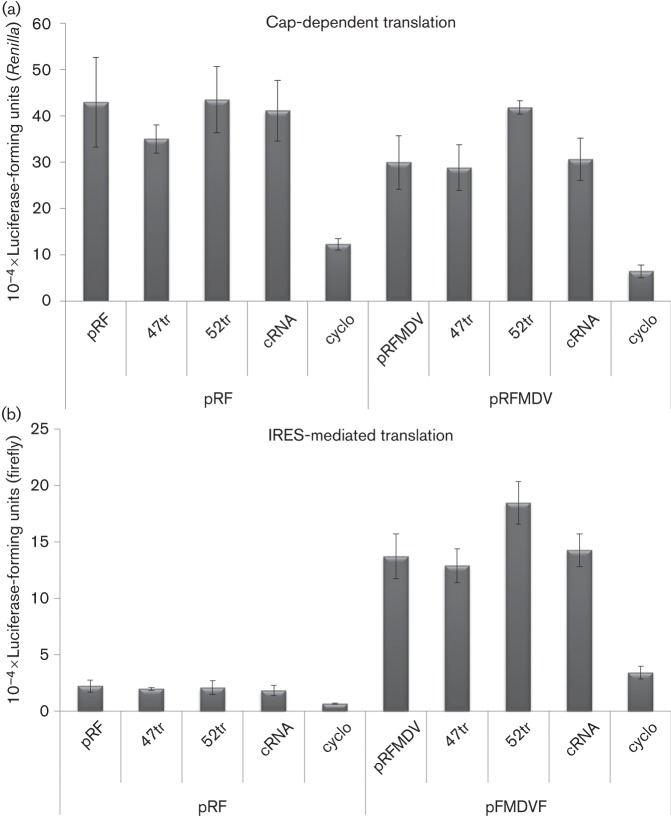
Effect of aptamers on translation. BHK-21 cells were transfected with a construct containing *Renilla* and firefly luciferase reporter genes under either (a) cap- or (b) FMDV IRES-mediated translation respectively (pRFMDVF). At 24 h post-transfection of the constructs, cells were subjected to a second transfection with aptamers 2′F-Cy3-47tr, 2′F-Cy3-52tr or cRNA. Cells transfected with the pGFP-PAC replicon and either subjected to a second mock-transfection or treated with 1 µg cycloheximide (cyclo) µl^−1^ were included as controls. Cells were lysed 24 h post-treatment and firefly and *Renilla* luciferase units (RLU) were measured using the dual-luciferase reporter assay system (Promega). A construct (pRF) containing only a cap-mediated *Renilla* luciferase reporter was included as a further control. Data are means±se of three experiments. Statistical analysis was conducted using a *t-*test (*P*<0.05).

Previously, we showed that, in addition to sharing similarity with each other, the aptamers also shared similarity with the viral genome (Ellingham *et al.*, 2006), particularly within the 5′UTR. To ensure that the effects of the aptamers seen here were related to replication and that the molecules were not affecting translation by 3′UTR or 5′UTR binding, we performed translation assays with bicistronic vectors. We employed a construct with *Renilla* and firefly luciferase reporter genes under cap- or FMDV IRES-mediated translation, respectively (pRFMDVF) (Licursi *et al.*, 2011). The data in Fig. 5 show expression of both firefly and *Renilla* luciferase from the pRFMDVF plasmid and showed that levels were unaffected by 2′F-Cy3-47tr, 2′F-Cy3-52tr or the cRNA (*P***>**0.05). A construct (pRF) containing only a cap-dependent reporter and cycloheximide (1 µg µl^−1^) were included as controls. For completeness, we also undertook similar experiments with constructs incorporating other viral IRES sequences (hepatitis C virus, human rhinovirus), with similar results (data not shown).

## Discussion

Subgenomic replicons have proved useful tools in the study of replication of a range of viruses, including hepatitis C virus (Lohmann *et al.*, 1999). Here, we use an FMDV replicon to study replication in BHK-21 cells in the presence of potential inhibitors. Protein expression (as monitored by GFP reporter expression) acts as a direct measure of replication (Tulloch *et al.*, 2014). Continual production of the RdRp 3D^pol^ facilitates replication of the replicon RNA to provide increasing levels of template for GFP expression, reaching maximal levels after approximately 8 h. This rapid expression of GFP is consistent with the fast replication rate of FMDV and is mirrored by a change in the morphology of the cells. It is likely that expression of the L^pro^ is responsible for this cytotoxic effect (Tulloch *et al.*, 2014).

We have demonstrated that previously characterized RNA aptamers selected against 3D^pol^ (the key enzyme in the replication complex) can be modified to increase their stability while maintaining effective inhibition of GFP expression. This inhibition is dose dependent, with maximal inhibition at 125–500 nM, which resulted in restoration of normal cell morphology. The aptamers were inhibitory at lower levels than seen with the control drug 2′CMC. However, it should be noted that the method of delivery of the drug and the aptamers (i.e. by transfection) is different. Further studies are ongoing in order to understand aptamer uptake and cellular localization.

It has been documented that chemically synthesized aptamers are non-toxic and non-immunogenic. The molecules used in this study were small (31–33 nt). They are predicted to possess minimal double-stranded regions (Doble *et al.*, 2014; Ellingham *et al.*, 2006) and do not include a 5′-triphosphate. It is therefore unlikely that they will trigger RIG-I/MDA5 IFN signalling (Belyaeva et al., 2014). We have shown that they are well tolerated in cells and that the aptamers are able to reverse the cytotoxic effects of replicon expression, with 2′F-Cy3-52tr being the most effective. The likely mode of action is via direct inhibition of 3D^pol^ activity, thus preventing the amplification of replication. There do not seem to be any effects on translation, although other off-target effects cannot be ruled out.

The first aptamer has been approved for clinical use (to treat macular degeneration; Ng et al., 2006), and several others are in clinical trials. Significantly, we have demonstrated that it is possible to deliver small aptamer RNAs passively to cells without the need for transfection reagents (Doble *et al.*, 2014), thus opening up possibilities for the therapeutic use of aptamers in a topical/localized manner. Furthermore, the inherent flexibility of RNA and conformational changes that occur upon target recognition open up possible applications for aptamers in diagnostics, alongside more conventional antibodies (reviewed by Brody & Gold, 2000). Therefore, the RNA aptamers described here, together with aptamers to other parts of the replication complex, are useful molecular tools in the study of replication but also have possible applications in diagnostics and therapeutics in the future.

## Methods

### 

#### Elongation assays.

Recombinant 3D^pol^ (WT or D388A mutant) was purified as described previously (Bentham *et al.*, 2012). Elongation assays were carried out using 2 µg poly(A) template (mean length 320) (Amersham), 2.4 µM oligo(dT)_15_ primer, 200 µM UTP, 5 µCi α-[^32^P]UTP and 3D^pol^ to a final concentration of 1 µM in binding buffer (30 mM MOPS, 10 mM NaCl, 25 mM magnesium acetate). For a time-course experiment, 10 µl samples were taken at time points up to 60 min and reactions stopped by addition of 10 µl 0.5 mM EDTA. Samples were spotted onto separate DE-81 filter papers (Whatman) and left to dry before washing five times with 200 ml 0.2 M Na_2_HPO_4_, then with 500 ml dH_2_O for 1 min and finally 100 ml 100 % ethanol for an additional minute. The filter papers were dried before adding to scintillation fluid for quantification of α-[^32^P]UTP incorporation by scintillation counting. For inhibition assays, 3D^pol^ (1 µM) was pre-incubated with 4 µM aptamer RNA for 20 min before addition to an elongation assay, as above.

#### Immunostaining of aptamer-transfected cells.

BHK-21 cells were employed as these are known to support vigorous FMDV replication. Cells grown on coverslips in 12-well dishes and transfected with 50 nM aptamer were washed with PBS for 2×2 min before fixing with ice-cold methanol for 10 min on ice. Cells were quickly washed once with PBS and permeabilized with ice-cold 50 % acetone/50 % methanol for 10 min and washed twice in PBS for 5 min. Coverslips were removed from wells and mounted onto glass slides in VectaShield plus DAPI (Vector Laboratories). Cells were imaged on an upright confocal microscope (Zeiss).

#### pGFP-PAC replicon RNA transcription.

A detailed description of the pGFP-PAC replicon cloning strategy and characterization is described elsewhere (Tulloch *et al.*, 2014). A further construct incorporating a deletion in 3D^pol^ was also described. To produce template for transcription, 1 µg plasmid DNA was linearized using 1 U *Hpa*I in final concentrations of 1× buffer 4 (NEB), 0.5 µg BSA in a 20 µl reaction and incubated at 37 °C for at least 4 h. Products were then analysed on a 1 % agarose gel and linearized vector was excised before gel purification. One microgram of linearized and purified vector DNA was transcribed into RNA in a 20 µl reaction using T*7* Megascript (Ambion) according to the manufacturer’s instructions. Each reaction was carried out for 3 h at 37 °C before treating with 1 U TurboDNase for 15 min at 37 °C. Samples were then phenol/chloroform extracted and ethanol precipitated. After ethanol precipitation, pellets were resuspended in 18 µl RNase-free water (Severn Biotech).

#### pGFP-PAC replicon RNA transfection.

The following mixture was prepared for each well of a 12-well plate: 1 µg pGFP-PAC replicon RNA was added to 250 µl OptiMEM (Invitrogen) and incubated at room temperature for 10–15 min. Complete Dulbecco’s modified Eagle’s medium (DMEM) was removed from the BHK-21 cells and replaced with the ESCORT reagent mixture (Sigma-Aldrich). The cells were incubated at 37 °C in a humidified CO_2_ incubator for up to 14 h. As appropriate, aptamer RNA (up to 500 nM) was added to the reaction pre-transfection. The control inhibitor, 2′-*C*-methylcytidine (2′CMC), was prepared in 0.5 % DMSO at a range of concentrations. At 30 min post-transfection, cells were washed twice in PBS prior to addition of 2′CMC (final concentration 0–50 µM).

#### Immunoblotting.

Cells were washed in PBS, lysed in lysis buffer (20 mM Tris pH 7.5, 10 % v/v glycerol, 1 mM EDTA, 150 mM NaCl, 1 % v/v Triton X-100) supplemented with Protease Inhibitor Cocktail (Roche) and incubated on ice for 5 min before clarification. SDS-PAGE was carried out using a 10 % gel system (Bio-Rad), followed by transfer of proteins to PVDF membrane (Whatman). 3D^pol^ was detected with polyclonal rabbit sera (kindly provided by Francisco Sobrino, Madrid) and secondary goat anti-rabbit HRP conjugate (Pierce). Cellular glyceraldehyde 3-phosphate dehydrogenase (GAPDH), employed as an internal control protein, was detected with an mAb (GAPDH-71.1, Sigma-Aldrich).

#### IncuCyte analysis of replication.

GFP expression and live cell imaging were analysed using IncuCyte FLR, an automated phase-contrast and fluorescent microscope within a 37 °C humidifying CO_2_ incubator. After transfection and co-transfection of subgenomic replicon RNA, cells were maintained in the IncuCyte and monitored every 30 min for up to 12 h. At different time points, nine images of each well were taken and GFP object count measurements analysed by using the integrated algorithm.

#### RNA synthesis.

Aptamers 2′F-Cy3-47tr and 2′F-Cy3-52tr (Elllingham *et al.*, 2006) and control cRNA were synthesized by phosphoramidite chemistry in house. cRNA is a previously reported aptamer selected to IL-17A, a non-viral protein (produced primarily by CD4^+^ lymphocytes) (Doble *et al.*, 2014).

The sequences of the aptamers used are as follows. Both 2′F-Cy3-47tr and 2′F-Cy3-52tr include 2′F C and cRNA includes 2′F C and U: 2′F-Cy3-47tr, 5′-Cy3-GGGUUAACAGAAAACCUCAGUUGCUGGGUUGU-3′; 2′F-Cy3-52tr, 5′-Cy3-GGCUUACCCGUGAACCGCAGUUGCUGAGUUG-3′; and cRNA, 5′-GGUCUAGCCGGAGGAGUCAGUAAUCGGUAGACC-3′.

#### Translation of bicistronic vectors.

BHK-21 cells (1×10^6^) were seeded in a 96-well plate culture plate and cultured overnight at 37 °C, 5 % CO_2_. The cells in each well were transfected with 0.125 µg pRF or pRFMDVF (Licursi *et al.*, 2011) plasmid in the presence of 31.25 µl OptiMEM and 0.625 µl ESCORT reagent. The plates were incubated overnight at 37 °C and 5 % CO_2_. At 24 h post-transfection, the cells were transfected again with one of the following RNA aptamers at 500 nM – 2′F-Cy3-47tr, 2′F-Cy3-52tr or cRNA – or treated with cycloheximide (Sigma) at 1 µg µl^−1^. After incubation for a further 24 h, the cells were lysed in 20 µl 1× Passive Lysis Buffer (Promega) and samples were assayed using dual luciferase assay reagents using Stop and Glow substrate in a 1 : 2 dilution according to the manufacturer’s instructions (Promega).
